# Recent Pharmacological Developments on Rhodanines and 2,4-Thiazolidinediones

**DOI:** 10.1155/2013/793260

**Published:** 2013-05-02

**Authors:** Ravinder Singh Bhatti, Sakshi Shah, Suresh, Pawan Krishan, Jagir S. Sandhu

**Affiliations:** ^1^Department of Pharmaceutical Sciences and Drug Research, Punjabi University, Patiala 147 002, India; ^2^Department of Chemistry, Punjabi University, Punjab, Patiala 147 002, India

## Abstract

Thiazolidines are five-member heterocyclic having sulfur, nitrogen, and oxygen atoms in their ring structure and exhibiting potent as well as wide range of pharmacological activities. In this minireview, recent updates on synthesis and pharmacological evaluations of molecules based on 2,4-thiazolidine and rhodanine are discussed.

## 1. Introduction

Five-membered heterocyclic molecules containing thiazole nucleus with carbonyl group on fourth carbon such as rhodanine and 2,4-thiazolidinedione derivatives have broad spectrum of pharmacological activities. In past two decades, rhodanines and 2,4-thiazolidinediones have emerged as potent antidiabetic agents. Some of them are clinically used such as ciglitazone, englitazone, pioglitazone, glitazones, epalrestat, and troglitazone for the treatment of type 2 diabetes mellitus and related complications. This is the reason why investigation/molecular modification and pharmacological evaluation of these molecules have attracted special attention of synthetic chemists and pharmacologists, respectively. 


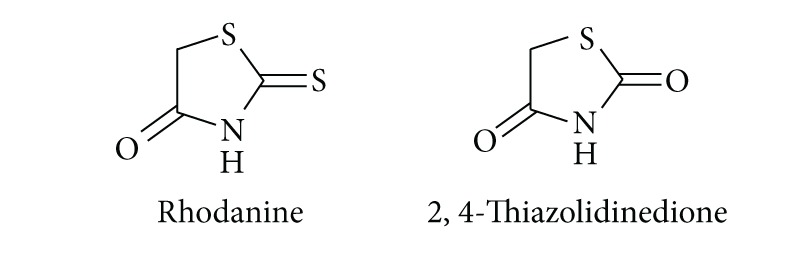


In recent years, a number of synthetic/pharmacological protocols based on these molecules have been emerged extensively and in witness available in the literature. These multifaceted molecules exhibit varied type of biological activities. Some recent developments in synthesis and pharmacology of these molecules are discussed in this section. 

## 2. Recent Developments in Rhodanine Pharmacology

In 1997, Boyd carried out a study based on rhodanine-containing molecules of pharmaceutical interest and found pharmacological importance of these molecules is limited because of poor solubility of rhodanine derivatives in water (exception of rhodanine-3-acetic acids). However, these compounds exhibit a broad range of significant biological activities [1]. Rhodanine-3-acetic acid (RAA) **1** was prepared by Korner [2] in 1908, and Knoevenagel condensation products of the acid with various aldehydes, namely, [(5*Z*)-(5-benzylidene-4-oxo-2-thioxo-1,3-thiazolidin-3-yl)]acetic acids **2** were reported in the same year [3]. From 1960 onwards, studies revealed that such type of molecule exhibit potential antimycobacterial [4, 5], antifungal [6–15], pesticidal [16–18], antihypertensive [19], and antineoplastic [20, 21] activities. Their NMR characterization performed in 1982 [22]. In 2006, similar derivatives have been prepared under microwave irradiation [23]. Further, the Knoevenagel products of rhodanine-3-acetic acid with pyridinecarbaldehydes were prepared in 1961 and they possess potential antibacterial and antifungal activities [8]. {(5*Z*)-[4-Oxo-5-(pyridin-2-ylmethylidene)-2-thioxo-1,3-thiazolidin-3-yl]}acetic acid **3** were patented as a potential drug for the treatment of metabolic bone diseases [24, 25]. Later, it was found out that they stimulate parathyroid hormone receptor-mediated cAMP formation and could be useful for the local and systemic treatment of rheumatoid arthritis, osteoarthritis, and degenerative arthrosis [24, 25].


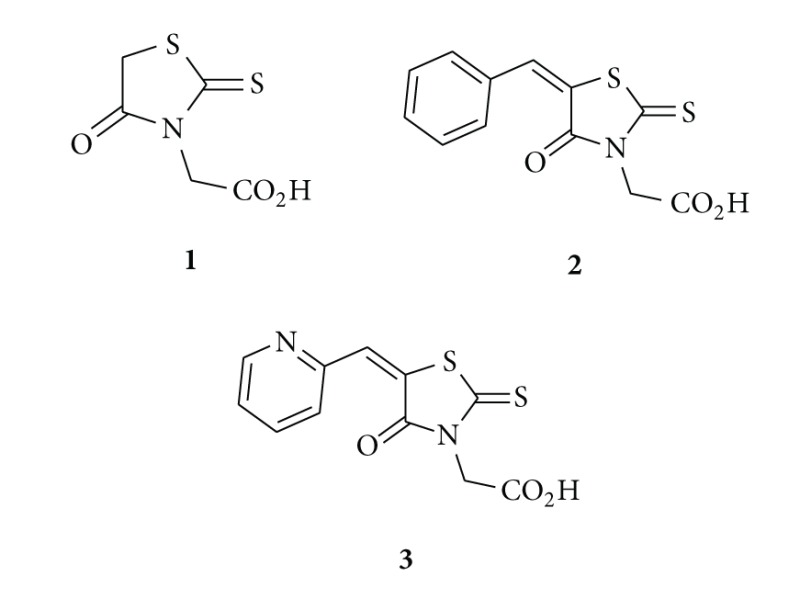


Trypanocidal activity of substituted rhodanine-3-acetic acids has been reported recently [26]. The only rhodanine acetic acid derivative that has been used clinically is the aldose reductase inhibitor epalrestat **4**. It was marketed in Japan and used to slow eye damage associated with diabetes and to prevent diabetic peripheral neuropathy [1, 22, 27–29]. Aldose reductase is not the only enzyme inhibited by rhodanine carboxylic acids. It was found that many other enzymes are also inhibited by the derivatives of this structural class and may be responsible for their various biological effects [30]. Other rhodanine-based molecules have also been popular as small molecule inhibitors of numerous targets such as hepatitis C viral (HCV) NS3 protease [31], antidiabetic mechanism [32], aldose reductase [33], *β*-lactamase [34, 35], histidine decarboxylase [36], and JNK Stimulatory Phosphatase-1 (JSP-1) [37]. This section is a brief account on synthesis and biological effects and recent developments of newly prepared potential drugs based on nitrogen-sulphur containing heterocycles having rhodanine nucleus.


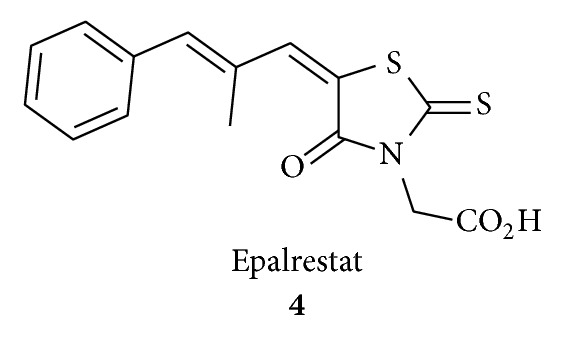


### 2.1. Rhodanine as Antidiabetic Agent

Murugana et al. synthesized [38] a series of dispiropyrrolidines **5** (16-compounds) by 1,3-dipolar cycloaddition reaction of azomethine ylides (*in situ* generated by the reaction of sarcosine with isatin) with 5-arylidene-1,3-thiazolidine-2,4-dione and 5-arylidene-4-thioxo-1,3-thiazolidine-2-one derivatives as dipolarophiles. They performed molecular docking studies on 1FM9 protein and screened synthesized compounds for their antidiabetic activity on male Wistar rats (after alloxan treatment). The synthesized compounds exhibited attractive antidiabetic properties and are more effective than rosiglitazone in ameliorating stress conditions.


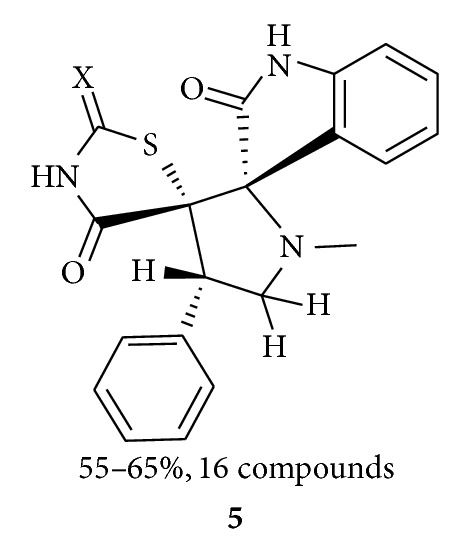


### 2.2. Rhodanine as Antiapoptotic Agent

Xing and his co-worker synthesized, a series of BH3I-1 based dimeric modulators of **6**. The overexpression of antiapoptotic Bcl-2 proteins which protects cells from apoptosis is one mechanism for tumours to acquire drug resistance. In this study they found dimeric modulators **7-8** have enhanced binding activity against antiapoptotic Bcl-2 proteins and proved dimerization of monomeric modulators is one practical approach to enhance the bioactivity of Bcl-2 antagonists [39]. 


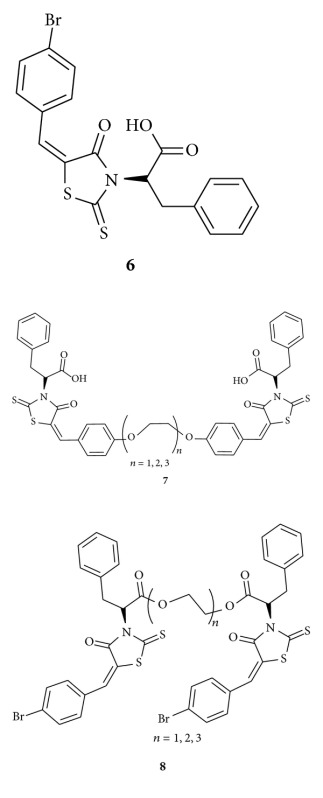


Moorthy and his group [40] designed and synthesized 5-isopropylidiene derivatives of 5-benzilidene-3-ethyl rhodanine (BTR-1) **9**, 3-dimethyl-2-thio-hydantoin (ITH-1) **10**, and 3-ethyl-2-thio-2,4-oxazolidinedione (ITO-1) **11** and tested their chemotherapeutic properties. They found all the compounds induced cytotoxicity in a time- and concentration-dependent manner on leukemic cell line, CEM. Among these compounds, BTR-1 **9** was found to be manifold more potent in inducing cytotoxicity than ITH-1 **10** and ITO-1 **11** with an IC50 value of <10 *μ*M and affected cell division by inducing a block at S phase, which finally led to the activation of apoptosis.


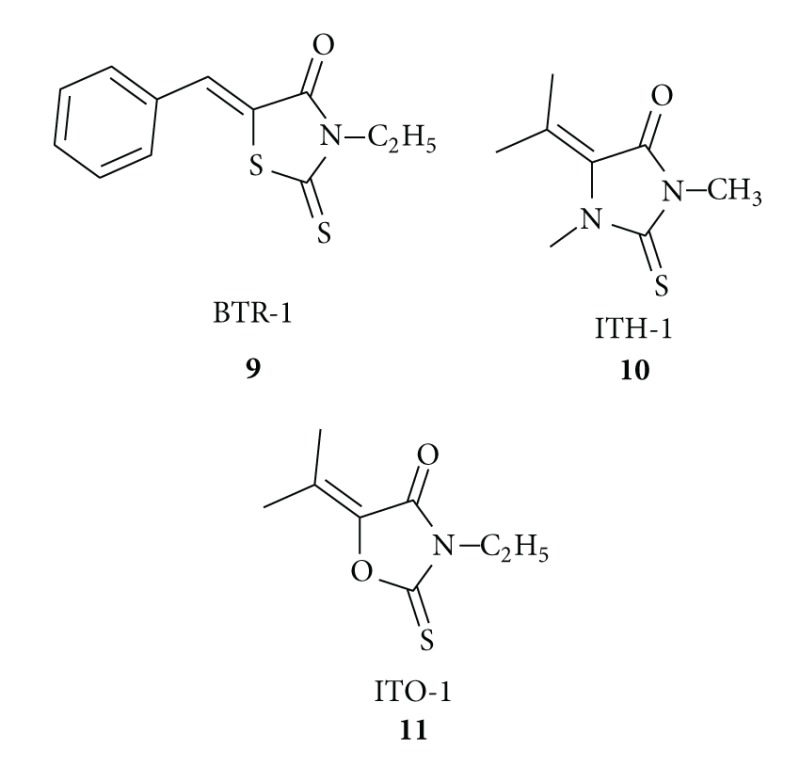


The same research group reported [41] the synthesis of 5-isopropylidene-3-ethyl rhodanine **12** by conventional and microwave-assisted method, and they found that rhodanine ITR **12** treatment led to cytotoxicity in leukemic cell line, CEM, by inducing apoptosis.


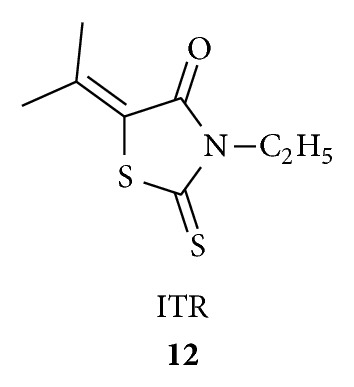


### 2.3. Rhodanine as Antimicrobial Agent

Habib et al. reacted [42] thiazolo[4,5-dlpyrimidines with rhodanines and investigated the obtained products (7 compounds with 60–80% yields) **13** for antimicrobial screening and they found antifungal activity against *Aspergillus niger* and *Penicillium* sp. with IZ 20–38 mm and MIC <50–<25 *μ*g/mL. They claimed compound **14** is the most active against *Aspergillus niger* while compound **15** is the most active against *Penicillium *sp.; and 5-fold less active than the standard antibiotic clotrimazole. They concluded that the presence of an alkyl group at position 3 of the thiazolopyrimidine ring **14** is superior to that of other aromatic substituents; also the introduction of an arylideneamino group at position 6 of **15** enhanced the antifungal activity.


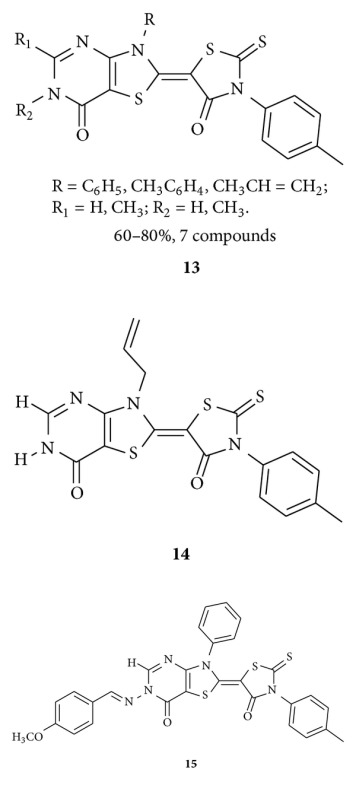


Opperman and his group disclosed [43] that aryl rhodanines **16–19** did not exhibit antibacterial activity against any of the bacterial strains tested and are not cytotoxic against HeLa cells. Their study revealed that the aryl rhodanines **16–19** specifically inhibit the early stages of biofilm development by preventing attachment of the bacteria (specifically inhibit biofilm formation of *S. aureus, S. epidermidis, Enterococcus faecalis, E. faecium,* and *E. gallinarum* but not the Gram-negative species *Pseudomonas aeruginosa* or *Escherichia coli.*) to surfaces.


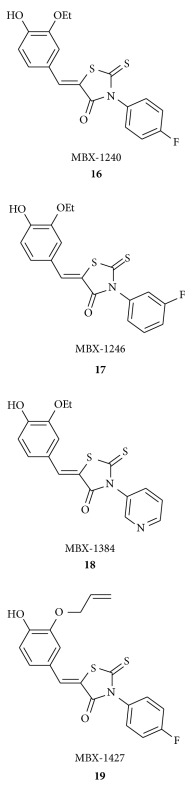


Sim and his associates reported [44] benzylidene rhodanines **20–22** as novel inhibitors of uridine diphospho-N-acetylmuramate/L-alanine ligase. They observed that compounds **20–22** exhibit selective whole-cell activity against the Gram-positive methicillin resistant *Staphylococcus aureus* (MRSA) but not against the Gram-negative *Escherichia coli*. They also evaluated their cytotoxic effect on mammalian Chinese hamster ovary (CHO) cells.


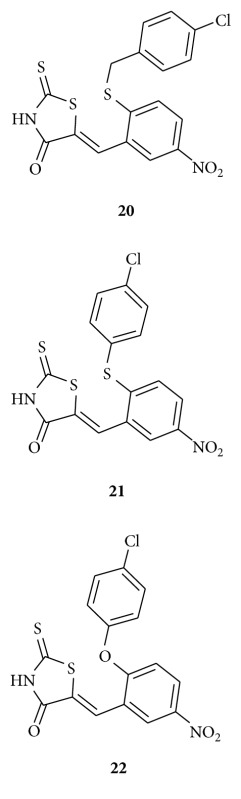


Hardej et al. synthesized [45] a series of glycine and phenylalanine-derived rhodanine analogs and evaluated their anti-MRSA activity. The antibacterial activity of compounds **23** and **24** against a panel of MRSA strains was significantly greater than that of the reference antibiotics penicillin G and ciprofloxacin. They claimed compound **24** exhibited only a 2–4-fold higher MIC value than that of vancomycin. They concluded from their study that the phenylalanine-derived compounds **23** and **24** are promising templates for the development of new drugs to treat MRSA infections.


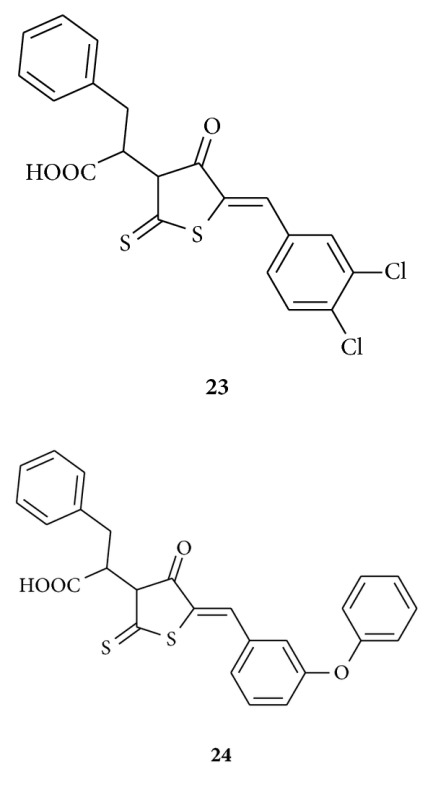


Chen and his group synthesized [46] several hybrid compounds (19 compounds with 31–58% yields) **25** having chalcone and rhodanine-3-acetic acid units and tested these compounds for their antibacterial activity. They found some compounds presented great antimicrobial activities against Gram-positive bacteria (including the multidrug-resistant clinical isolates) as active as the standard drug (norfloxacin) and less active than oxacillin. 


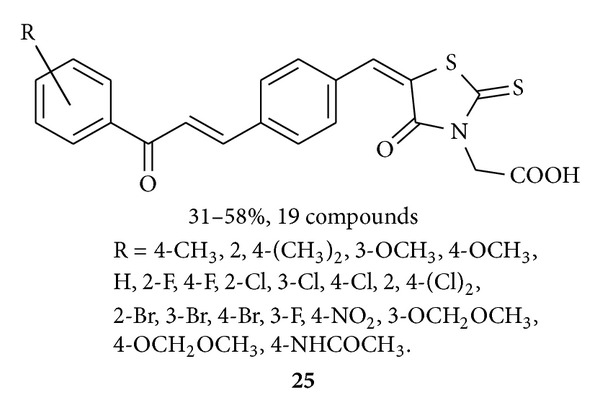


Tomasic et al. reported [47] the synthesis and antibacterial activity for a series of rhodanine-, rhodanine-N-acetic acid-, thiazolidine-2,4-dione-,barbituric-, and thiobarbituric acid-based compounds bearing an ylidene substituent at position 5. The most potent compound of the series, (*Z*)-5-(2,3,4-trifluorobenzylidene)rhodanine **26**, inhibited the growth of *S. aureus* at 0.5 mg/mL and MRSA at 32 mg/mL. 


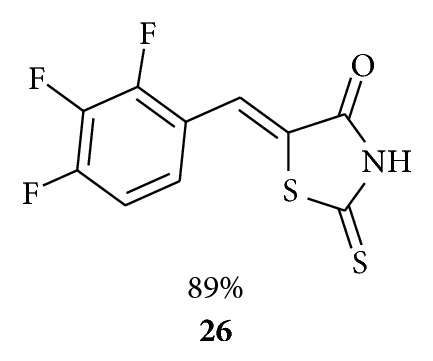


Orchard and his group [48] synthesized rhodanine-3-acetic acid-based compounds **27-28** and described as inhibitors of fungal protein: mannosyl transferase 1 (PMT1). They observed 5-[[3-(1-phenylethoxy)-4-(2-phenylethoxy)phenyl]methylene]-4-oxo-2-thioxo-3-thiazolidineacetic acid **29** inhibit *Candida albicans* PMT1 with IC50s in the range 0.2–0.5 *μ*M. Members of the series are effective in inducing changes in morphology of *C. albicans in vitro* that have previously been associated with loss of the transferase activity. According to them, these compounds **27-28** could serve as useful tools for studying the effects of protein O-mannosylation and its relevance in the search for novel antifungal agents.


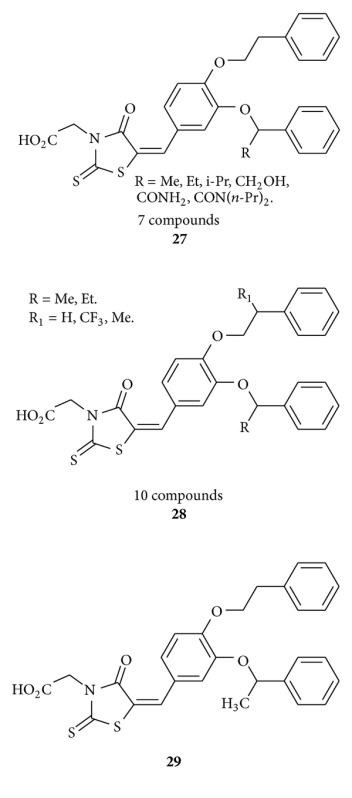


Sortino et al. reported [49] benzylidene-rhodanines **30** which act as antifungal agents. They evaluated that compounds **31** and **32** showed fungicidal activity and are the most active against *Candida genus* and *C. neoformans* including clinical isolates. Other compounds of this series showed a very good activity against dermatophytes.


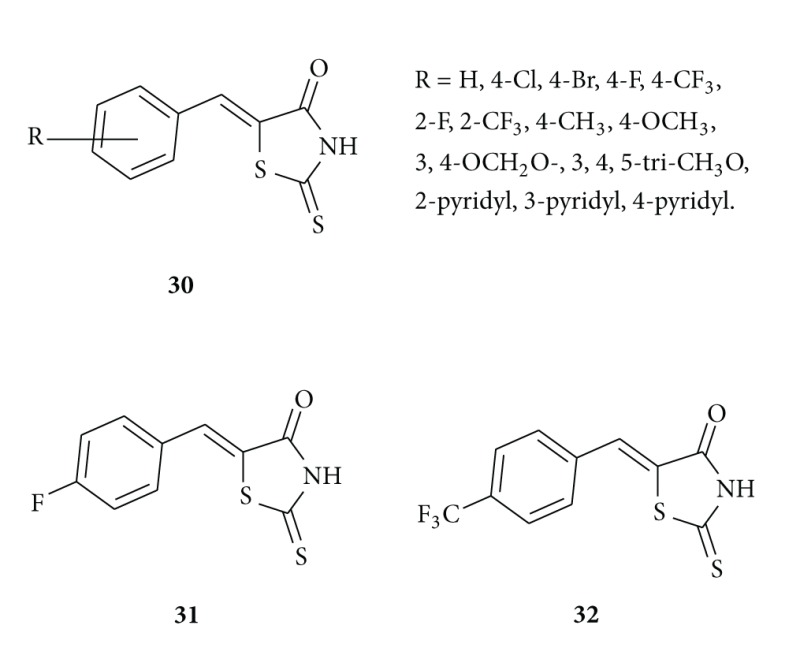


### 2.4. Rhodanine as Antihepatitis C Virus (HCV) Agent

Sing et al. disclosed [50] arylalkylidene rhodanines **33-34** inhibit HCV NS3 protease at moderate concentrations. They claimed these rhodanine derivatives are better inhibitors of serine proteases such as chymotrypsin and plasmin. They concluded that selectivity of arylmethylidene rhodanines **33-34** with bulkier and more hydrophobic functional groups increases by 13- and 25-fold towards HCV NS3 protease, respectively.


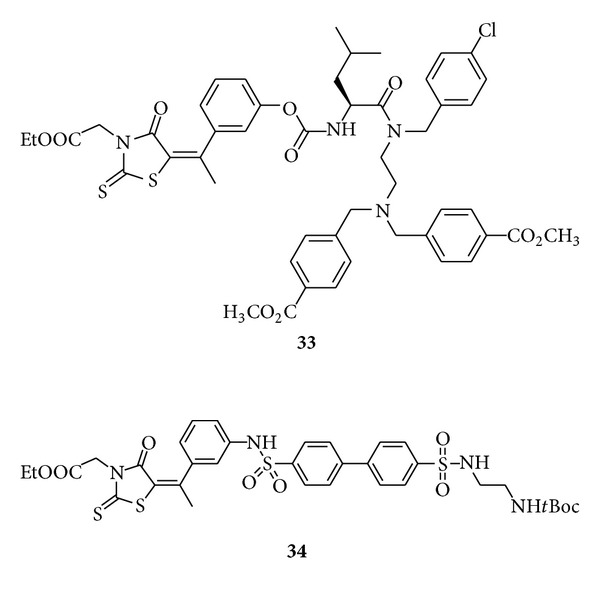


### 2.5. Rhodanine as HIV-1 Integrase Inhibitors

Rajamaki and his associate synthesized [51] and biologically evaluated rhodanine-based compounds **35** and identified these exhibiting anti-HIV-1 integrase activity and moderate inhibition of HIV-1 cell replication. 


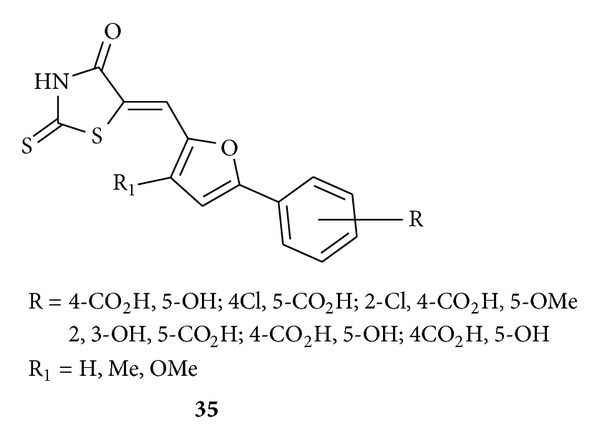


### 2.6. Rhodanine as Anti-Inflammatory Agent

Cutshall et al. reported [52] synthesis and evaluation of rhodanine-based compounds **36** as inhibitors of JSP-1. On SAR studies they demonstrated that stronger electron-withdrawing functional groups appended to the aryl-benzylidene position provided analogs with the greatest potencies as illustrated by compound **37**. Compound **37** has also reversible and competitive bind with substrate and showed a high degree of enzyme selectivity against other phosphatases. 


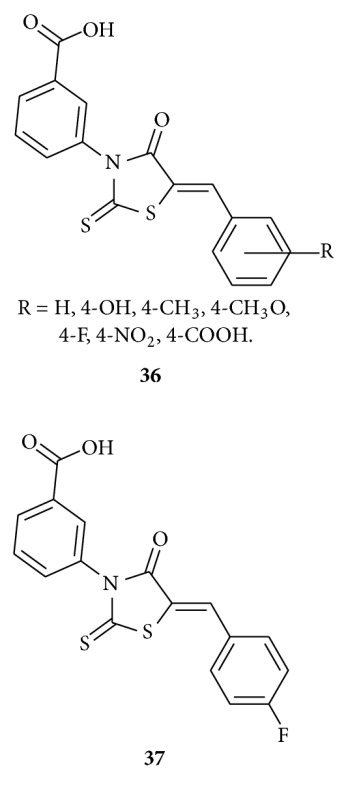


Irvine et al. identified [53] a series of rhodanine derivatives as novel inhibitors of phosphodiesterase type 4 (PDE4). Structures **39** and **40** displayed the most significant activity of the compounds synthesized, being some 20- and 24-fold more potent than lead compound **38**. 


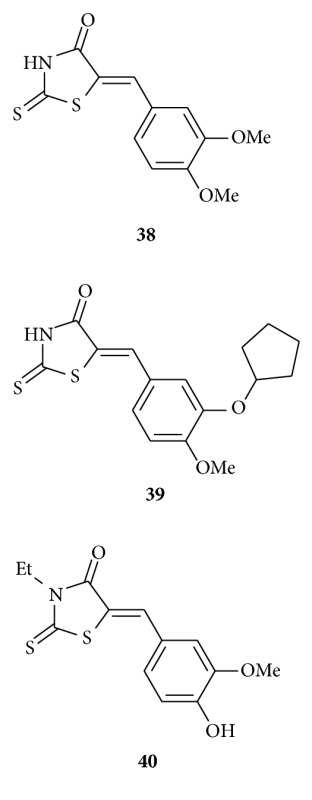


### 2.7. Rhodanines for Sleeping Sickness

Smith et al. developed [26] the first small molecular inhibitors of dolicholphosphate mannose synthase (DPMS), a mannosyltransferase critically involved in glycoconjugate biosynthesis in *T. brucei*. They claimed thiazolidinones **41**, **42,** and **43** in particular are promising candidates for further development because of their respective activities against trypanosomal DPMS and GPI anchor biosynthesis. They reported that these DPMS inhibitors prevent the biosynthesis of glycosylphosphatidylinositol (GPI) anchors and possess trypanocidal activity against live trypanosomes. Drug-like molecules **41–43** with activity against *Trypanosoma brucei* are urgently required as potential therapeutics for the treatment of African sleeping sickness. 


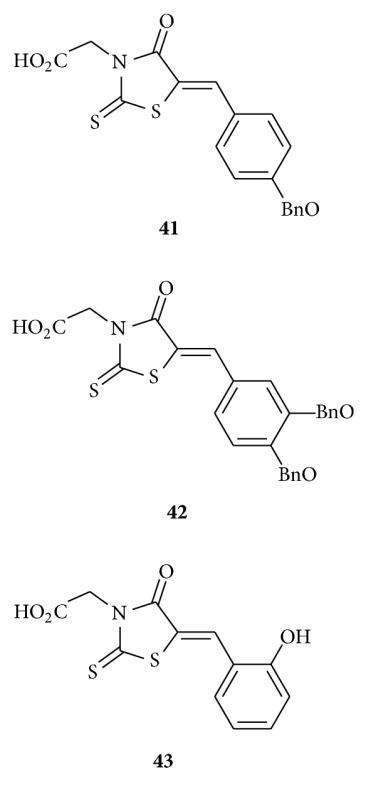


### 2.8. Rhodanines as Tyrosinase Inhibitors

Liu et al. synthesized [54] a series of dihydropyrimidin-(2H)-one analogues and rhodanine derivatives and evaluated their inhibitory effects on the diphenolase activity of mushroom tyrosinase. In results they found that some of the synthesized compounds exhibited significant inhibitory activities. Particularly, compound **44** bearing a hydroxyethoxyl group at position-4 of phenyl ring exhibited the most potent tyrosinase inhibitory activity with IC50 value of 0.56 mM. The inhibition mechanism analysis of compound **44** demonstrated that the inhibitory effect of the compound on the tyrosinase was irreversible. These results suggested that such compounds might be served as lead compounds for further designing new potential tyrosinase inhibitors.


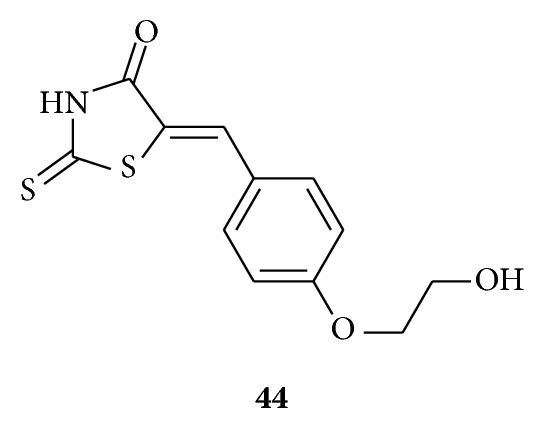


### 2.9. Rhodanines as PRL-3 Inhibitors

Ahn et al. synthesized [55] and evaluated a series of rhodanine derivatives **45** for their ability to inhibit oncolytic phosphatase (PRL-3). Benzylidene rhodanine derivative **45** showed good biological activity, while compound **46** is found to be the most active in this series exhibiting IC50 value of 0.9 Lm *in vitro* and showed a reduced invasion in cell-based assay. 


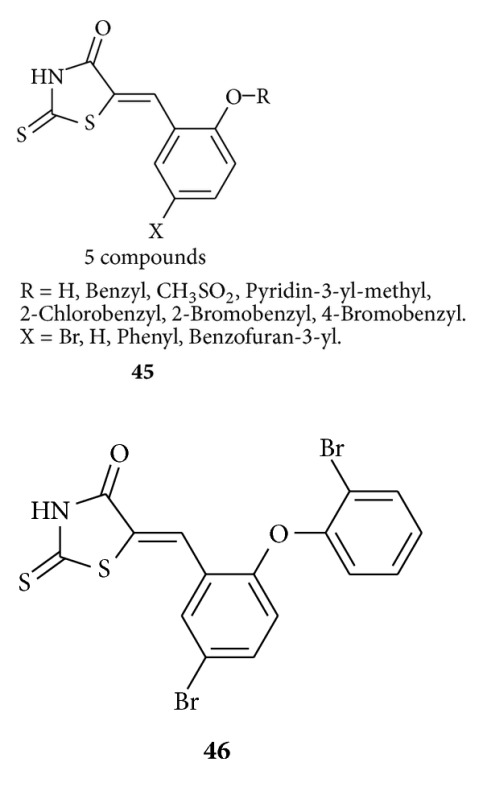


## 3. Pharmacological Developments in 2,4-Thiazolidinediones

The most commonly used antidiabetic agents have been sulfonylureas, metformin, and certain alphaglucosidase inhibitors and meglitinides. These agents increase insulin secretion from pancreatic *β*-cells but sometimes induce severe hypoglycemia and weight gain [56], and hyperinsulinemia is known to be a risk factor for ischemic heart disease [57]. In addition, high rates of both primary and secondary failure have been observed with these drugs [58–61]. Therefore, drugs that ameliorate the insulin resistance without stimulating insulin release from *β*-cells have been developed for the treatment of type 2 diabetes. Type 2 diabetes is a multifactorial disease defined by a high plasma glucose level and is characterized by both insulin resistance and impaired insulin secretion by pancreatic *β*-cells [62]. The prototypical 2,4-thiazolidinedione, ciglitazone **47** was discovered [63] by Takeda Chemical Industries, Ltd., Japan, and has antihyperglycemic activity in insulin-resistant animal models, KKAy mice [64], and Wistar fatty rats [65] but no effect in insulin-deficient animal models of diabetes [66, 67]. During structure-activity relationship studies on 2,4-thiazolidinediones and related compounds, they discovered highly potent compounds, such as pioglitazone **48** [68] and AD-5061 **49 **[69]. Since the discovery of ciglitazone **47**, a number of pharmaceutical companies have been evaluating new 2,4-thiazolidinedione analogs as agents for improving insulin resistance. Troglitazone **50 **[70] was launched first in the market but had been withdrawn because of liver toxicity and related deaths associated with the drug. Nowadays, two 2,4-thiazolidinedione class agents, pioglitazone **48** and rosiglitazone **51 **[71], have been clinically used. Furthermore, many companies are still endeavoring to find a new glucose-lowering agent [72–84]. 


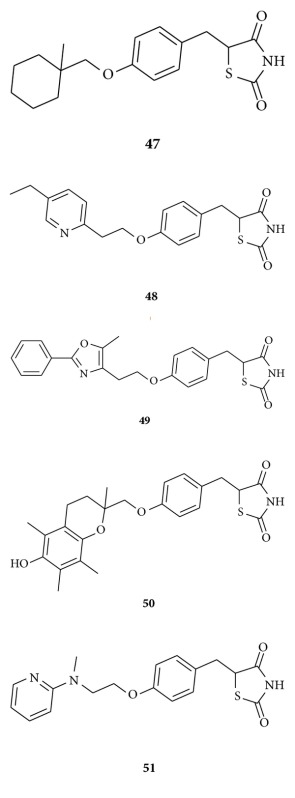


Although the precise mechanism of action of these drugs remains unknown, a recent study suggests that antidiabetic thiazolidinediones interact with a family of nuclear receptors known as peroxisome proliferator-activated receptor (PPAR)-*γ* [85]. PPAR*γ* is one of a subfamily of PPARs encoded by independent genes. Three human PPARs, designated PPAR*α*, PPAR*γ*, and PPAR*δ*, have been identified to date [86–88]. It was also observed that the potency for the activation of PPAR*γ* *in vitro* mirrored the *in vivo* glucose-lowering activity in diabetic *ob/ob* mice [89]. This would indicate that the major mechanisms of action of 2,4-thiazolidinediones involve PPAR*γ*. In case of those 2,4-thiazolidinediones already on the market, several side effects, such as anaemia, edema, and body weight gain, have been reported [90]. Therefore, search for new compounds with fewer side effects and a more advanced profile than existing drug molecules is the main focus of attention for chemists as well as for pharmacologists. Recent developments in the synthesis and evaluations of thiazolidinedione-based compounds for a variety of biological activities along with antidiabetes is discussed in the ongoing pages. 

### 3.1. Thiazolidinedione as Antidiabetic Agent

Rakowitz et al. synthesised [91] and tested several 5-benzyl-2,4-thiazolidinediones **52-53** as *in vitro* aldose reductase inhibitors (ARIs). Their evaluation shows N-unsubstituted 5-benzyl-2,4-thiazolidinediones **52** and (5-benzyl-2,4-dioxothiazolidin-3-yl)acetic acids **53** displayed moderate-to-high inhibitory activity levels. The insertion of an acetic chain on N-3 significantly enhanced AR inhibitory potency, leading to acids **53** which proved to be the most effective among the tested compounds. In addition, in N-unsubstituted derivatives **52** the presence of an additional aromatic ring on the 5-benzyl moiety was generally beneficial. 


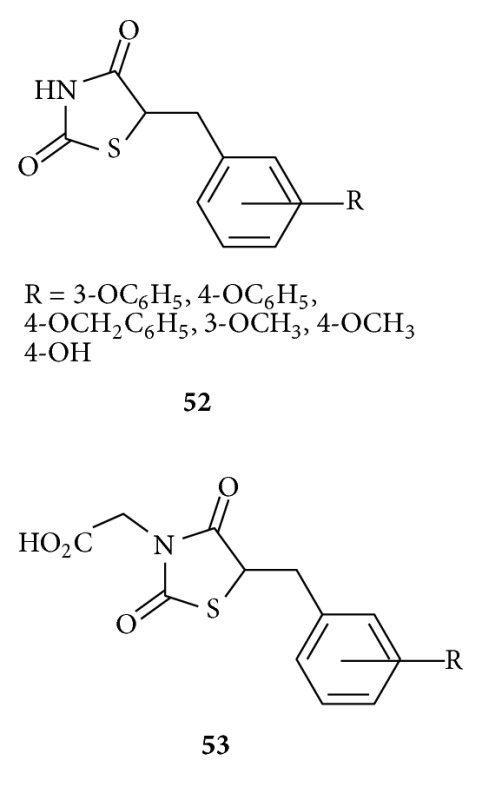


Madhavan et al. synthesized [92] and evaluated 2,4-thiazolidinedione derivatives of 1,3-benzoxazinone for their PPAR-*α* and -*γ* dual activation. They obtained that a compound DRF-2519 (**54**), through SAR of TZD derivatives of benzoxazinone, has shown potent dual PPAR activation. In *ob/ob* mice, it showed better efficacy than the comparator molecules. In fat fed rat model, it showed significant improvement in lipid parameters, which was better than fibrates.


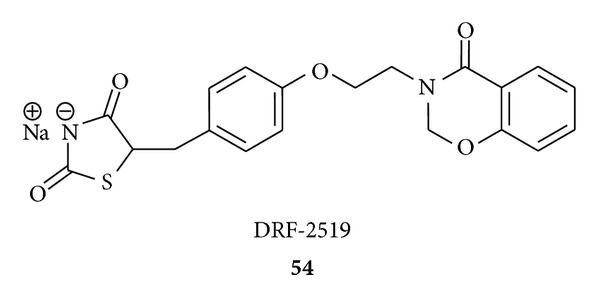


Bozdağ-Dündar et al. prepared [93] a new series of chromonyl-2,4-thiazolidinediones **55** aiming to reduce diabetic complications especially which have effect on the cataract formation. The synthesized compounds were tested for their ability to inhibit rat kidney AR by an *in vitro* spectrophotometric assay. Compound **56** showed the highest inhibitory activity. They concluded that the increasing inhibitory effect of compounds **55** might depend on the acetic acid side chain of 2,4-thiazolidinedione, and these compounds especially **56** could display therapeutic potential in the prevention and the treatment of diabetic complications as promising ARIs.


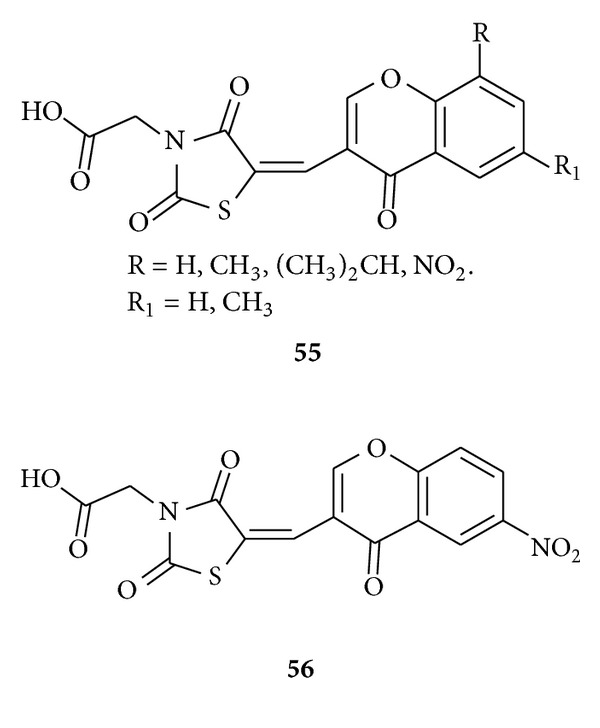


Maccari et al. reported [94] new ARIs via *in vitro* evaluation of a series of 5-arylidene-3-(3,3,3-trifluoro-2-oxopropyl)-2,4-thiazolidinediones **57** as ARIs allowed the identification of two new noncarboxylic acid containing 5-arylidene-2,4-thiazolidinedione derivatives (**58** and **59**) that found active at low-micromolar doses. 


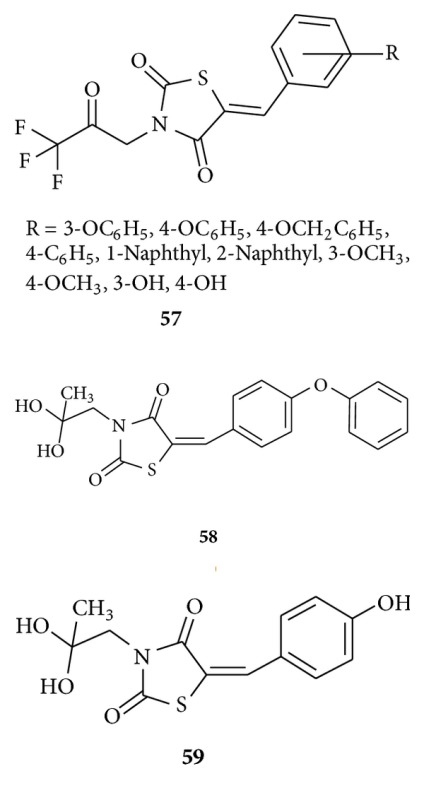


### 3.2. Thiazolidinedione as Anticancer Agent

Patil et al. synthesized [95] and evaluated ten derivatives of 5-benzylidene-2,4-thiazolidinediones **60** for their antiproliferative activity in a panel of 7 cancer cell lines using four concentrations at 10-fold dilutions. Sulforhodamine B (SRB) protein assay used to estimate cell stability or growth. These compounds showed varying degrees of cytotoxicity in the tested cell lines, most marked effect observed by compound **60** in MCF7 (breast cancer), K562 (leukemia), and GURAV (nasopharyngeal cancer) cell lines with log10 GI50 values of −6.7, −6.72, and −6.73, respectively.


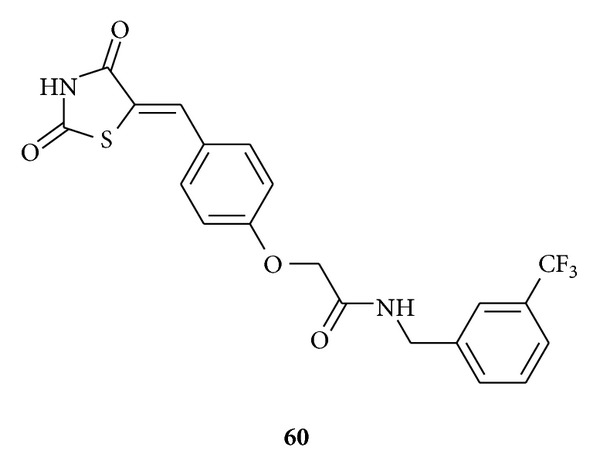


### 3.3. Thiazolidinedione as Anti-Inflammatory Agent

Barros et al. synthesized [96] 5-arylidene-3-benzyl-thiazolidine-2,4-diones **61** with halide groups on their benzyl rings (8 compounds) and assayed in vivo to investigate their anti-inflammatory activities, and 3-(2-bromo-benzyl)-5-(4-methanesulfonyl-benzylidene)-thiazolidine-2,4-dione, compound **62**, showed higher anti-inflammatory activity than the rosiglitazone reference drug as it bound PPAR*γ* with 200-fold lower affinity than the reference ligand. 


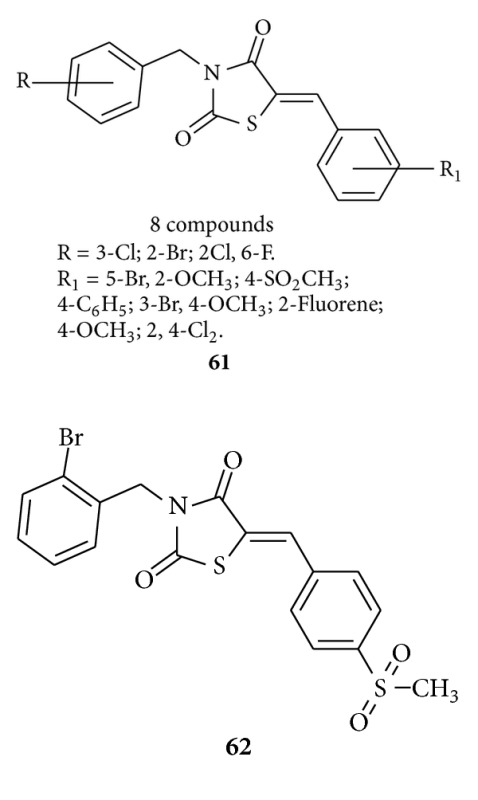


Alagawadi and Alegaon described [97] the synthesis and antimicrobial activity of 5-substituted-2,4-thiazolidinedione derivatives. They evaluated all compounds for their preliminary *in vitro* antibacterial and antifungal activity. The investigation of antimicrobial screening revealed that some of the tested compounds showed moderate to good bacterial and fungal inhibition. Particularly, compounds **63–66** have shown good activity against *S. aureus* and *E. faecalis* with minimum inhibitory concentration (MIC) values between 4 and 32 *μ*g/mL. They found all compounds active against tested fungal strains at 1–64 *μ*g/mL concentration. Compounds **63-66** also showed good antifungal activity against *C. albicans* at 1–4 *μ*g/mL and *C. neoformans, A. flavus, A. niger* at 2–8 *μ*g/ml concentration. 


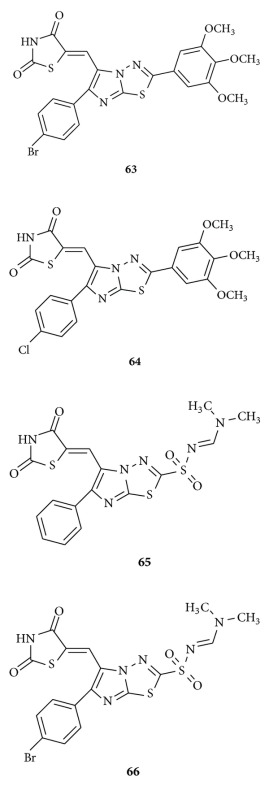


Liu et al. synthesized [98] a series of chalcone derivatives bearing the 2,4-thiazolidinedione and benzoic acid moieties **67** and evaluated for their antibacterial activity. In tested compounds, the most effective results obtained with MIC value in the range of 0.5–4 mg/mL against six Gram-positive bacteria (including multidrug-resistant clinical isolates). 


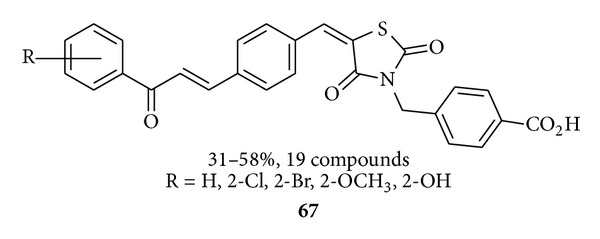


### 3.4. Thiazolidinedione as Antioxidant

Jeong et al. synthesized [99] multisubstituted benzylidenethiazolidine-2,4-diones **68** by Knoevenagel condensation of di- or trisubstituted 4-hydroxybenzaldehydes with thiazolidine-2,4-dione and evaluated the antioxidant activities of Cu^2+^-induced oxidation of human low-density lipoproteins (LDLs). Among compounds, **69** was found to be superior to probucol in LDL-antioxidant activities and found to be 9-fold more active than probucol.


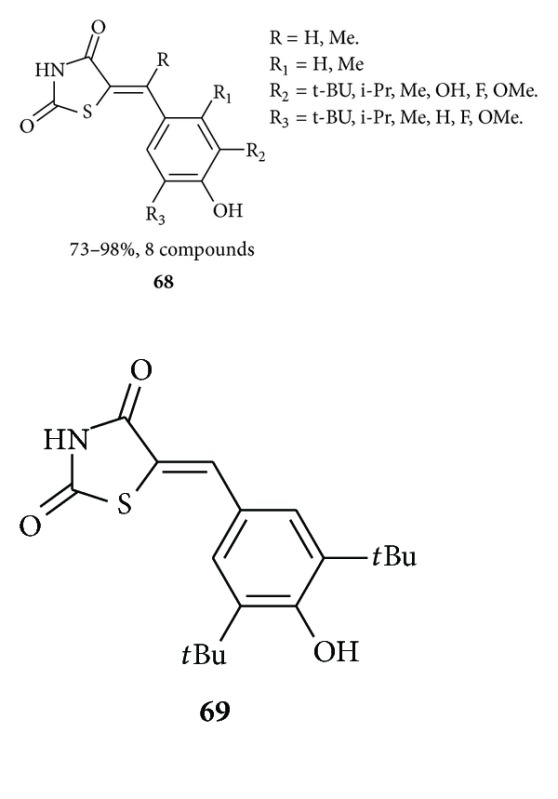


Hossain and Bhattacharya synthesized [100] a series of 5-arylidene-2,4-thiazolidinediones and its geranyloxy or prenyloxy derivative and studied their radical scavenging activity using 1,1-diphenyl-2-picrylhydrazyl (DPPH) assay. They expressed comparable scavenging activities as IC50 value. Compounds **70–72** showed appreciable radical scavenging activities. The vanillin-based thiazolidinedione compound **70** displayed the highest activity comparable to that of *α*-tocopherol. But in vivo, compound **72** showed better results in inducing phase II detoxifying/antioxidative enzyme. The compounds **70–72** found to be effective in enhancing the host antioxidant defence system such as superoxide dismutase (SOD), catalase (CAT), glutathione-S-transferase (GST), and reduced Glutathione (GSH) and at the same time lowering the serum ALT and AST level at the preliminary screening dose of 3 mg/kg in normal Swiss albino mice given orally for 20 days as compared to the control animals. The hepatic lipid-peroxidation level (LPO) remained unchanged.


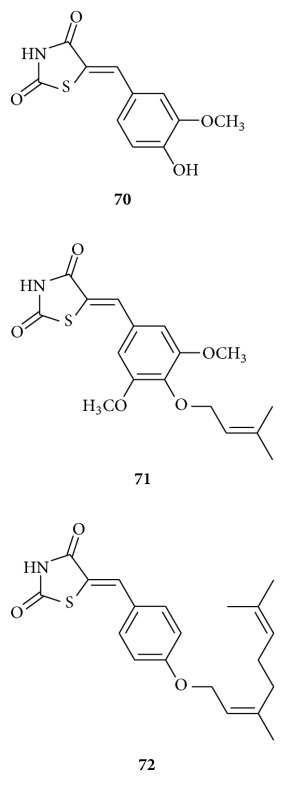


Ottanà et al. explored [101] 5-arylidene-4-thiazolidinones as antioxidant agents and aldose reductase inhibitors. They found that compounds **73** and **74** proved to be interesting inhibitors of the enzyme as well as excellent antioxidant agents that are potentially able to counteract the oxidative stress associated with both diabetic complications as well as other pathologies. 


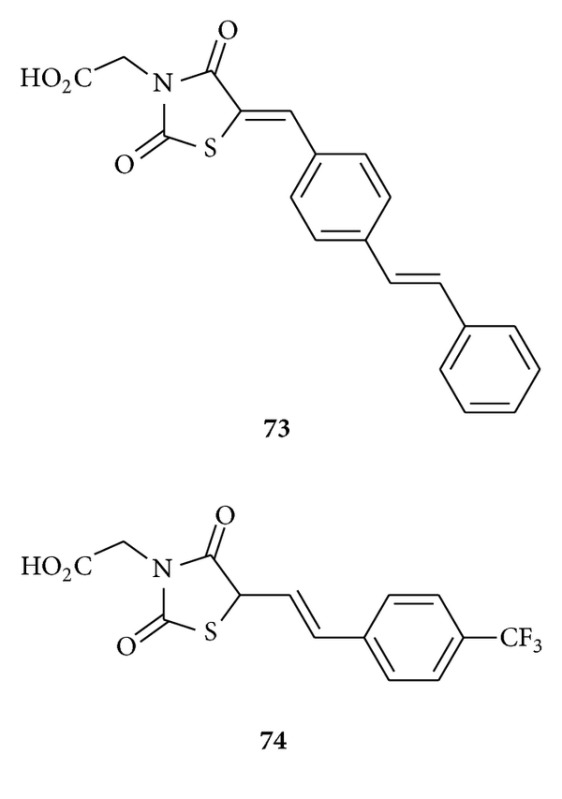


### 3.5. Thiazolidinedione as Antiobesity

Hu et al. disclosed [102] synthesis of methylsulfonamide-substituted 2,4-thiazolidinedione (6 compounds) **75** and found **76** to be a potent (EC50 = 0.01 mM, IA = 1.19) and selective (more than 110-fold over *β*1 and *β*2 agonist activity) *β*3 agonist. This compound has also been proven to be active and selective in an *in vivo* mode. 


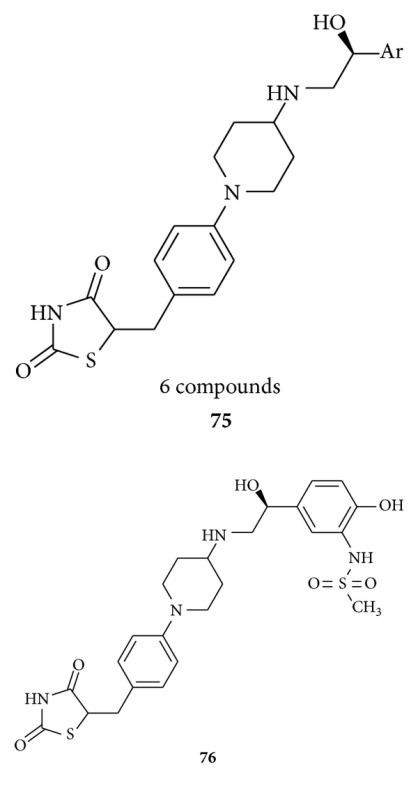


Bhattarai et al. synthesized [103] benzylidene-2,4-thiazolidinedione derivatives (9 compounds) with substitutions on the phenyl ring at the ortho or para positions of the thiazolidinedione group **77** as protein tyrosine phosphatase (PTP1B) inhibitors with IC50 values in a low-micromolar range. Compound **78**, the lowest, bores an IC50 of 5.0 *μ*M. In vivo efficacy of **78** as an antiobesity and hypoglycemic agent evaluated in a mouse model system. This compound also significantly suppressed weight gain and significantly improved blood parameters such as TG, total cholesterol, and NEFA. Compound **78** also was found to activate peroxisome proliferator-activated receptors (PPARs) indicating multiple mechanisms of action.


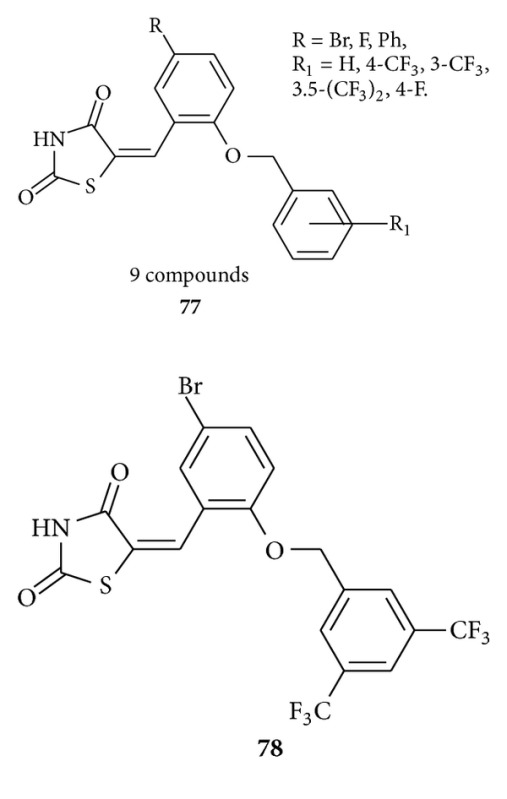


Same research group [104] synthesized benzylidene-2,4-thiazolidinedione derivatives (12 compounds) **79** as PTP1B inhibitors with IC50 values in a low-micromolar range. Compound **80**, the lowest, bores an IC50 of 1.3 *μ*M. In a peroxisome proliferator-activated receptor-*γ* (PPAR-*γ*) promoter reporter gene assay, **80** was found to activate the transcription of the reporter gene with potencies comparable to those of troglitazone, rosiglitazone, and pioglitazone. In vivo efficacy of **80** as an antiobesity and hypoglycemic agent was evaluated in a mouse model system. Compound **80** significantly suppressed weight gain and significantly improved blood parameters such as TG, total cholesterol, and NEFA without overt toxic effects.


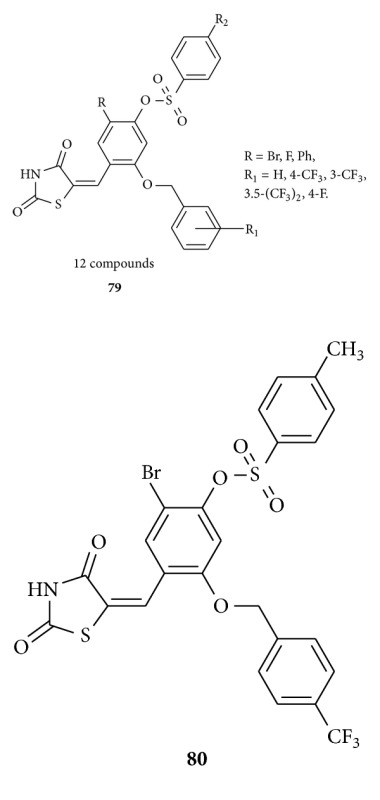


### 3.6. Thiazolidinedione as Antiprostaglandins

Ying et al. synthesized [105] a range of benzylidene thiazolidinedione derivatives (27 compounds with 75–88% yields) with different substituents on the phenyl ring and evaluated their inhibitory 15-hydroxyprostaglandin dehydrogenase (15-PGDH) activity. Based on the structures of the thiazolidinediones analogues and inhibitory activity, replacement of the cyclohexylethyl group of **81** with the hetero five-member ring increased the inhibitory potency. However, replacement of the cyclohexylethyl group with a hetero six-member ring decreased the inhibitory potency significantly. They found that compound **82** (5-(4-(2-(thiophen-2-yl)ethoxy)benzylidene)thiazolidine-2,4-dione) is the most potent inhibitor and effective in the nanomolar range.


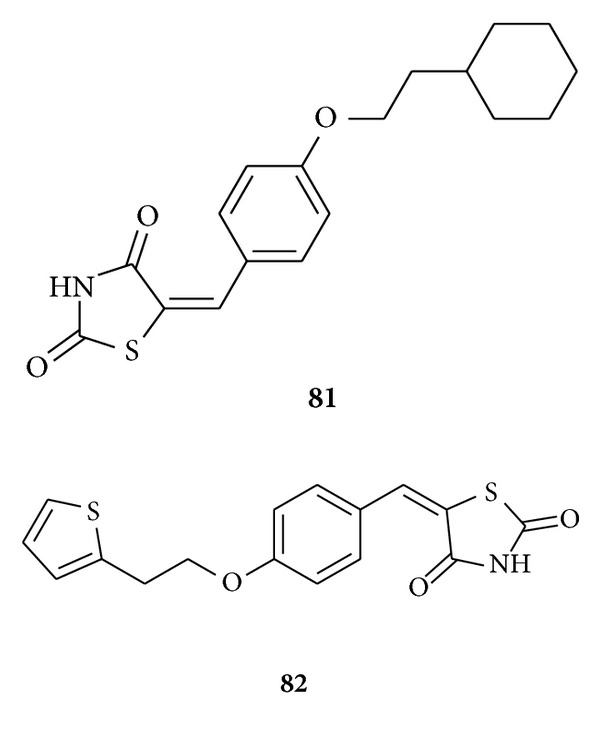


### 3.7. Thiazolidinedione as Thyroid Hormone Receptor Antagonists

Komatsu et al. designed and synthesized [106] diphenylamine derivatives **83** with a thiazolidinedione moiety as the terminal polar group as thyroid hormone receptor (TR) antagonists. Thiazolidinedione derivatives **83** with N-alkyl group showed antagonistic activities towards both the hTR*α*1 and hTR*β*1 subtypes.


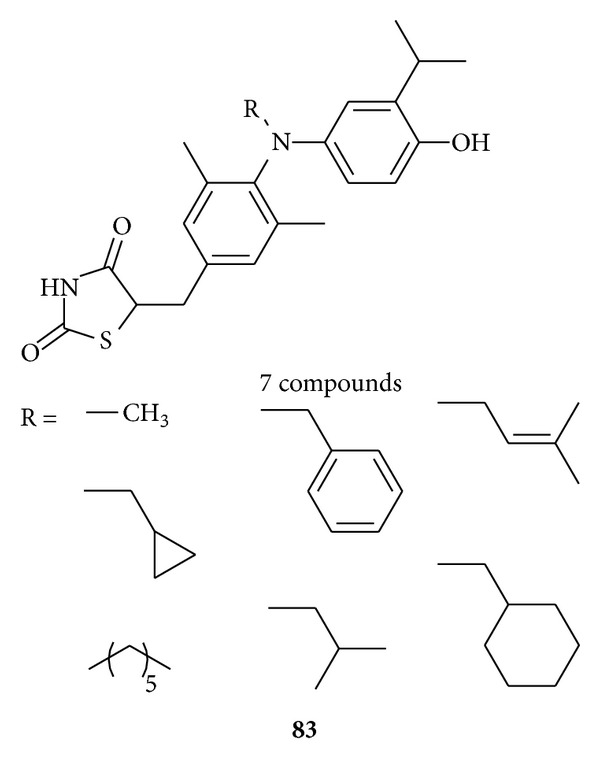


## 4. Conclusion

In recent past, a variety of molecules based on rhodanine and thiazolidinedione have been synthesized and evaluated with improved pharmacological activities. Due to wide range of pharmacological activities and clinically used 2,4-thiazolidines, these molecules have attracted much attention and encouraged the chemists and biologists to be extensive investigations or molecular manipulations, and as a result further improved protocol with better observation is still under progress. 
